# What works with men? A systematic review of health promoting interventions targeting men

**DOI:** 10.1186/1472-6963-8-141

**Published:** 2008-07-03

**Authors:** Lynn M Robertson, Flora Douglas, Anne Ludbrook, Garth Reid, Edwin van Teijlingen

**Affiliations:** 1 University of Aberdeen, Department of Public Health, Polwarth Building, Foresterhill, Aberdeen, AB25 2ZD, UK; 2 University of Aberdeen, Health Economics Research Unit, Polwarth Building, Foresterhill, Aberdeen, AB25 2ZD, UK

## Abstract

**Background:**

Encouraging men to make more effective use of (preventive) health services is considered one way of improving their health. The aim of this study was to appraise the available evidence of effective interventions aimed at improving men's health.

**Methods:**

Systematic review of relevant studies identified through 14 electronic databases and other information resources. Results were pooled within health topic and described qualitatively.

**Results:**

Of 11,749 citations screened, 338 articles were assessed and 27 met our inclusion criteria. Most studies were male sex-specific, i.e. prostate cancer screening and testicular self-examination. Other topics included alcohol, cardiovascular disease, diet and physical activity, skin cancer and smoking cessation. Twenty-three interventions were effective or partially effective and 18 studies satisfied all quality criteria.

**Conclusion:**

Most of the existing evidence relates to male sex-specific health problems as opposed to general health concerns relevant to both men and women. There is little published evidence on how to improve men's uptake of services. We cannot conclude from this review that targeting men works better than providing services for all people. Large-scale studies are required to help produce evidence that is sufficiently robust to add to the small evidence base that currently exists in this field.

## Background

Over the past decade, men's health has increasingly become a public health concern. Whilst their health has been improving over time, men still have a lower life expectancy than women. With the exception of Alzheimer's disease, men had higher mortality rates than women for the 15 leading causes of death in the US in 1999 [1]. Moreover, there are marked health inequalities between men living under different social circumstances [2].

Encouraging men to make more effective use of health services is considered one means of improving men's health, since there are significant differences in the way men and women seek help about health concerns [3]. Men tend to visit their doctor later in the course of a condition than women and this has been associated with poorer health outcomes [4,5]. Banks [5] also argues that better use would be made of existing health services if these were more "male friendly" in terms of convenience and anonymity.

It has been suggested that men have a very functional view of their bodies and see health care as a 'fix-it' cure; therefore they may respond better to health care interventions that offer tests, facts and figures, e.g. cholesterol, blood pressure [6,7]. The importance of female partners' support and influence on men's health behaviours has also been highlighted [8,9]. Thus, a potentially effective way to change men's behaviour, particularly their use of primary care, may be to target women who act as motivators of help-seeking behaviours among men [1]. Some authors have also advocated more proactive approaches in efforts to encourage men to use health care services earlier, such as outreach services [4,10].

There have been several well men's health initiatives introduced in the UK [11], but their effectiveness is not known. Previous reviews have focused on interventions aimed at reducing sexual risk behaviour among men [12-18]. Other than the area of sexual health, we know of no systematic reviews of specifically designed interventions aimed at men. This systematic review aimed to appraise the available evidence of effective interventions aimed at improving men's health.

## Methods

### Identifying relevant studies

Systematic searches were conducted in a wide range of electronic databases and other information resources to locate both published and unpublished studies in the English language from 1990 to April 2006. Only English language studies were included because of lack of resources. Studies were sought by systematic searching of three key journals: *Journal of Men's Health and Gender, Men's Health Journal *and *The International Journal of Men's Health*; 14 electronic databases; men's health websites; scanning reference lists of already identified relevant studies; and through personal contacts. A search strategy was devised for use with MEDLINE and adapted for other databases (see Additional file 1). Two independent reviewers assessed articles for inclusion and extracted data using standardized data abstraction forms. Any discrepancies were resolved by consulting a third reviewer.

### Criteria for considering studies for this review

Primary studies evaluating the effectiveness of interventions targeting men were included. These interventions could be directed either at improving specific health outcomes or improving men's uptake of services.

Studies were selected according to the following criteria: (a) participants: healthy, free-living adult men ≥ 18 years; (b) intervention: aimed at improving men's health; (c) setting: conducted in industrialised/developed countries; (d) outcomes: health status, knowledge, attitudes, behaviour, behaviour intentions; and (e) study design: randomised controlled trials (RCTs), quasi-randomised controlled trials (quasi-RCTs, non random allocation), controlled before-and-after studies. Studies were excluded if they concerned sexual health only, as several reviews already existed in this area.

### Quality Assessment

Studies were classified according to four criteria: (1) employing an appropriate control group; (2) providing pre-intervention data for all individuals; (3) providing post-intervention data for all individuals; and (4) reporting findings for each outcome measure indicated in the aims of the study [17]. When commenting on the studies, account has also been taken of the size of the study samples and the generalisability of the populations studied.

## Results

### Literature identification, study characteristics and quality

Of 11,749 citations screened, 338 articles were assessed and 27 met our inclusion criteria (see Table 1). Eighteen studies were RCTs, three quasi-RCTs and the remaining six were controlled before-and-after designs. Seventeen studies (63%) were male sex-specific, i.e. prostate cancer screening and testicular self-examination. Other health topics included alcohol, cardiovascular disease, diet and physical activity, skin cancer and smoking cessation. There were no specific studies on drug use or mental health. In three studies, the interventions were designed to improve attendance rates. The majority were in primary care (67%) and US-based (59%). In just over half of the studies, a theoretical framework guided the development and/or delivery of the intervention. Twenty-three interventions were effective or partially effective and 18 studies satisfied all quality criteria.

**Table 1:** Characteristics of Included Studies

**Author Setting Participants**	**Study Design Outcomes**	**Intervention Control/Comparison**
**Smoking Cessation**		
**Jenkins et al. 1997** Community, USA n = 1411 18+ years Vietnamese speaking	Controlled before-and-after 1. Proportion of current smokers 2. Proportion who had quit smoking	Intervention: Media-led anti-tobacco campaign San Francisco. Over 2 years Control: Comparison community.
**Pallonen, U. E. et al. 1994** Primary care, Finland n = 375 42–60 years	RCT 1. 7 day point prevalence abstinence. 2. Prolonged abstinence 3. Probability of stage changes 4. Exposure and subject evaluation of intervention	Intervention: Self help manuals 2 years Control: Usual care
**Stanton et al. 2004** Primary care, Australia n = 561 16–56 years Partners are pregnant	RCT Quit rate at end of pregnancy	Video/NRT and information/support material. Control: Brochure providing contact details for the available smoking cessation options.

**Diet and Physical Activity**		
**Cook et al. 2001** Workplace, New Zealand n = 253 mean 35 years at intervention site mean 42.9 years at control site male employees	Controlled before-and-after 1. Dietary and lifestyle behaviours 2. Nutrition knowledge 3. BMI 4. Waist circumference 5. BP	Intervention: Health promotion programme – 30 min workshop once a month for 6 months Control: No treatment
**Williams and Lewis 2002** University, USA n = 45 20–25 years, mean 21.5	RCT 1. Percent calories from fat 2. Assessment of counselling	1. Nutrition counselling and measurement of cholesterol (NC + SC) 2. Nutrition counselling only (NC) 3. Measurement of serum cholesterol only (SC) Control: No intervention.

**Cardiovascular Disease**		
**McCrone et al. 2001** Primary care, USA n = 33 57–78 years	Controlled before-and-after 1. Body composition 2. Metabolism 3. CV fitness	Intervention: SM: Multibehavioural stress management (nutrition, exercise and stress management) Control: ED: Multibehavioural educational intervention (nutrition, exercise and education) 6 months Exercise physiologist, dietitian and nurse.
**Pritchard et al. 2002** Workplace, Australia n = 66 mean 43.4 years overweight men	RCT 1. Weight 2. Fat and lean mass 3. Dietary energy and percentage dietary fat intake 4. Physical activity indices 5. BP 6. Insulin, blood lipids and lipoproteins	1. Dietary intervention – low-fat diet using the National Heart Foundation booklet, The Weight Loss Guide 2. Exercise intervention – subjects selected own exercise for a min 3 × 30 mins p/week above their pre-study exercise level (12 months) Control: Maintain pre-study dietary and activity patterns (16 of control group followed a diet and exercise program for a subsequent 12 months).

**Prostate Cancer**		
**Davison et al. 1999** Primary care, Canada n = 100 50–79 years, mean 62.1	RCT 1. Preferred and assumed roles in screening decision making 2. Levels of state anxiety 3. Levels of decisional conflict 4. Screening behaviour	Intervention: Verbal and written information about the controversies surrounding screening for prostate cancer. Control: Investigator talked about general issues.
**Flood et al. 1996** Primary care, USA Study 1: n = 409 Study 2: n = 222 50+ years	Quasi RCT 1. Knowledge concerning prostate cancer and screening 2. Preference regarding treatment and screening	Study 1 Intervention: Educational video Control: Control video Study 2 Intervention: Educational video Control: No intervention
**Frosch et al. 2003** Primary care, USA n = 226 50+ years	RCT Primary outcomes: 1. Convenience, effort required, satisfaction with intervention. 2. Knowledge 3. Choice of PSA test Also secondary outcomes.	Intervention: Prostate cancer education website Control: Prostate cancer education video
**Gattellari and Ward 2005** Community, Australia n = 421 50–70 years	RCT 1. Knowledge 2. Views towards PSA screening 3. Decisional conflict (post-test only) 4. Decisional control 5. Worry 6. Perceived ability to make informed choice 7. Propensity to undergo PSA screening during next 12 months 8. Likelihood of accepting doctor's recommendation to go to PSA screening (post test only) 9. Scenario-based assessment 10. Perceptions of GP fault regarding adverse consequences of screening decisions 11. Demographic and health info 12. Evaluation of materials received (post test).	Comparing 3 educational resources 1. Leaflet 2. Video 3. Booklet
**Hammond et al. 2001** Primary care, USA n = 1959 mean 66.5 years	RCT, cluster randomisation 1. Health Status 2. Urinary symptoms 3. Treatment received 4. Prostate related knowledge 5. Physicians management of prostate conditions	Intervention: Practice intervention for physicians. Patient brochures, 2 videotapes. 18 months Control: Usual care
**Myers et al. 1999** Primary care, USA n = 413 40–70 years African American	RCT Adherence (to come for education and early detection)	Intervention: Print materials and telephone contacts. "Pro-record" tailored to each recipient – educational booklet. Control: Print material and telephone contacts.
**Myers et al. 2005** Primary care, USA n = 242 ≥ 40 years African American	RCT Digital rectal examination (DRE) Prostate specific antigen (PSE)	Intervention: Enhanced Intervention (EI) Information book and screening decision educational session. Control: Standard Intervention (SI) Information Booklet
**Partin et al. 2004** Primary care, USA n = 1152 ≥ 50 years	RCT 1. Screening knowledge 2. Decision-making participation 3. Preferences 4. Behaviours	1. Mailed pamphlet 2. Mailed video No intervention
**Ruthman and Ferrans 2004** Primary care, USA n = 104 51–77 years, mean 66	Controlled before-and-after, staged 1. Knowledge 2. Preference for PSA test 3. Satisfaction with care 4. Assessment of Video	Intervention: Educational video "The PSA Decision: What you Need to Know". 20 minutes. Control: Usual care.
**Schapira and Van Ruiswyk 2000** Primary care, USA n = 257 50–80 years	RCT 1. Prostate cancer screening knowledge 2. Prostate cancer screening beliefs 3. Prostate cancer screening decisions (post-test)	Intervention: Illustrated pamphlet (decision-aid). Control: Written pamphlet.
**Summer et al. 2002** Workplace, England n = 458	Controlled before-and-after 1. Knowledge 2. Intentions to seek help 3. Attitudes to health promotion in the workplace 4. Process indicators	Site 1: Posters and leaflets Site 2: Posters and leaflets, visit by nurse Site 3: Posters and leaflets, visit by nurse and team of 8 men's health volunteers
**Volk et al. 2003** Follow-up of Volk et al. 1999 at 1 year Primary care, USA n = 160 45–70 years	RCT 1. Screening behaviours 2. Satisfaction with screening decision 3. Knowledge of prostate cancer	20 minute educational video on advantages and disadvantages of PSA screening and accompanying brochure. Control: No intervention
**Volk et al. 1999** Primary care, USA n = 160 45–70 years	RCT 1. Knowledge of prostate cancer 2. Reported preferences for PSA testing 3. Ratings of the videotape	20 minute educational video on advantages and disadvantages of PSA screening and accompanying brochure. Control: No intervention.
**Weinrich et al. 1998** Primary care, USA n = 1717 40 – 70 years, mean 52	Quasi, 2 by 2 factorial, randomly assigned Participation in free prostate cancer screening	1. Traditional 2. Peer-educator 3. Client-navigator 4. Combination of peer-educator and client-navigator
**Wilt et al. 2001** Primary care, USA n = 550 50+ years	RCT 1. Knowledge about early detection and treatment 2. PSA testing	Intervention: "Early Prostate Cancer" pamphlet mailed 1 week before clinic appointment. Control: Usual care.

**Testicular Cancer**		
**McCullagh et al. 2005** Workplace and leisure sites, UK n = 835 15–44 years	Controlled before-and-after 1. Knowledge 2. Intention to perform TSE Practice of TSE	Intervention: Check'Em Out information resources at workplace and leisure sites. Control: No intervention
**Steadman and Quine 2004** University, England n = 159 18–35 years	RCT 1. Performance of TSE 2. Future intention to perform TSE	Intervention: Implementation intentions (Illustrated leaflet containing detailed instructions on how to perform TSE.) Control: Illustrated leaflet only.

**Preventive Health Screening**		
**Holland 2005** Primary Care, USA n = 5677 40–60 years	RCT 1. Colorectal cancer screening 2. Cholesterol screening 3. Prostate cancer screening 4. Preventive health care visits	Intervention Matrix: Health provider stickers, letter/pamphlet, loved-one postcard.

**Skin Cancer**		
**Youl et al. 2005** Primary Care, Australia n = 1322 30–79 years	RCT Rates of attendance	Intervention: Personalised letter plus information brochure Control: Personalised letter

**Alcohol**		
**Karlsson et al. 2005** Community, Finland N = 4418 30–49 years	Controlled before-and-after. 1. Drinking behaviour (AUDIT) 2. Annual alcohol consumption	Intervention: Self help pamphlet Control: No intervention

AUDIT = Alcohol Use Disorders Identification Test, BMI = body mass index, BP = blood pressure, CV = cardiovascular, DRE = digital rectal examination, NRT = nicotine replacement therapy, PSA = prostate-specific antigen, RCT = randomised controlled trial, TSE = testicular self-examination.

### Smoking cessation

Three studies of effective smoking cessation interventions were found. Two were RCTs; one using self-help manuals [19] and one using video and nicotine replacement therapy plus other support materials [20]. The third study was a controlled before-and-after comparison of a media-led anti-tobacco campaign [21]. All studies showed a higher quit rate in the intervention group but relied heavily on self-reported smoking status. Pallonen *et al*. [19] reported seven-day abstinence rates of 10% (intervention) and 6% (control) after two years. Stanton *et al*. [20] reported quit rates at the end of the partner's pregnancy of 16.5% (intervention) and 9.3% (control) and validated their results with carbon monoxide readings for a sub-sample. Jenkins *et al*. [21] found that smoking prevalence fell by 2.2% points over two years in the target area and increased by 1.3% points in the control area.

### Diet and physical activity

Two studies, aimed at improving diet and physical activity were identified [22,23] both showing the interventions to be partially effective. One RCT was small (n = 45) and compared a combination of nutrition counselling (NC) and serum cholesterol measurement (SC) interventions with no intervention. The NC+SC group showed significant reductions in fat intake over a six-week period (3.2%) based on self-reported food intake over 24 hour periods [22]. Cook *et al*. [23] reported on a workplace intervention, consisting of a monthly health promotion workshop. Significant self-reported changes were made in vegetable intake, physical activity and dietary knowledge compared with a control site. Changes in fruit intake, and eating breakfast were not significant. There was a significant reduction in systolic blood pressure (BP) but no difference was found for diastolic BP, body mass index (BMI) or waist circumference.

### CVD risk factors

There was considerable overlap in approach between studies aimed at cardiovascular risk factors and those aimed at diet and physical activity. A small RCT (n = 66) at a workplace in Australia found that low fat diet and/or exercise interventions were effective, although no one strategy showed significant benefit over the other [24]. Another small controlled before-and-after study compared stress management (n = 25) and education (n = 8) when combined with nutrition and exercise interventions [25]. This study showed stress management to be more effective on a number of variables.

### Prostate cancer

This was the largest group of studies comprising 11 RCTs [26-36], two quasi RCTs [37,38] and two controlled before-and-after studies [39,40]. Most studies concerned the provision of education about screening and treatment options. A range of interventions, including verbal information, written information and videos, were shown to be effective in raising levels of knowledge and increasing the involvement of men in decision making about screening. In some studies, particularly in the US, this led to reductions in demand for screening.

### Testicular cancer

Two studies regarding testicular self-examination (TSE) were identified [41,42]. In one study, the intervention group was instructed to formulate specific plans for when and where they would perform TSE [41]. In the intervention group, self reported TSE was significantly higher than in the control group (65% versus 40%) but there was no difference between the groups in future intention to perform TSE. In another study [42], information resources were provided at workplace and leisure sites. In the intervention group there was a significant increase in knowledge and performing TSE.

### Preventive Health Screening

One US study [43] evaluated the effectiveness of patient and/or physician interventions (including targeting partners) to increase men's utilization of preventive healthcare services (annual health assessment, colorectal cancer screening, prostate cancer screening and cholesterol screening). Men in the intervention groups were more likely to receive preventive healthcare office visits, cholesterol screenings and prostate cancer screenings. None of the interventions had a significant impact on the number of men who received colorectal cancer screenings.

### Skin Cancer

One RCT addressed the issue of screening for skin cancer among men in Australia [44]. The study assessed the impact of two methods of encouraging men to attend skin screening clinics, a personalised letter or the letter plus an additional brochure. Overall, there was no difference in rates of attendance between the groups. The addition of health information through the use of a glossy brochure increased rates of attendance among younger men (30 to 49 years).

### Alcohol

One Finnish study assessing alcohol consumption was identified [45]. This controlled before-and-after study evaluated the impact of a self-help pamphlet to support self-control of drinking. Overall there were no significant changes in drinking behaviour or consumption. Among "risky male drinkers" there was a significant reduction in Alcohol Use Disorders Identification Test (AUDIT) scores at the second follow-up, but no change in the level of drinking.

## Discussion

The review established that despite the relatively emergent status of men's health, a fair amount of literature exists on the topic as 338 articles were identified during the literature search. However, a large proportion described the setting-up and functioning of well-man programmes, and did not include formal evaluations relating to outcomes; as a result only 27 articles were included in the final review.

The majority of studies were concerned with decision-making processes and knowledge about prostate cancer screening. This is not surprising due to the considerable debate around the effectiveness of prostate cancer screening. The UK National Screening Committee [46] recommended that prostate cancer screening should not be introduced and that men should not be invited for prostate-specific antigen (PSA) testing in the way that women are invited for mammography.

The literature suggests that interventions may be more likely to be effective if they are theory-based [47]. In just under half of the studies in our review, a theoretical framework guided the development and/or delivery of the intervention. These included the transtheoretical model, transtheoretical cognitive-behavioural social learning model, decision-making theory, preventive health model and implementation intentions. Decision-making theory was most widely used within prostate cancer education interventions.

One study in this review assessed whether the addition of cholesterol measurement would increase the effectiveness of nutrition counselling in male university students [22]. Results indicated that the effectiveness of nutrition counselling was slightly increased when combined with cholesterol measurements, although measurement of cholesterol alone was not shown to be effective.

Only one study targeted men's partners [43]. Results showed that communicating with a man's loved one combined with a reminder system for providers was associated with increase in preventive healthcare screenings. The importance of family and friends in mediating health service usage is also stressed in the literature. This is not simply because the pressure from peers leads them to do it "but rather that they can maintain face or keep their male identity intact, by claiming to be pressurised into it" [48:113]. However, such indirect targeting may be seen as controversial, regardless of this evidence from the literature.

Four studies were workplace-based [23,24,40,42]. The workplace is increasingly being used as a setting to gain access to men for the provision of health information and consultation with a health professional [49]. However, this can only benefit men in employment, and not those who are arguably more in need, eg homeless, unemployed.

Excluding the studies relating to sex-specific interventions, there were only three specifically designed to be suitable for men only [20,43,44] and a fourth intervention was delivered at a manufacturing worksite that was male only [23]. In the study by Stanton and colleagues [20], one component of the intervention was a video introduced by a national football personality focusing on becoming a father and on the passive smoking health risks for the newborn. To communicate directly with the targeted male, Holland and colleagues [43] created personalised letters that focused on the recommended preventive health screenings necessary for men in that age range. The letters also indicated the importance of establishing a relationship with a doctor. Addressing the issue of skin cancer screening, Youl and colleagues [44] developed promotional material that included a letter of invitation signed by a popular Australian sportsman, and a brochure highlighting the tendency of men to delay having a skin examination and the fact that men, more frequently than women, died from melanoma.

Many of the interventions were sex specific, rather than gender sensitive, i.e. they aimed to prevent diseases unique to men, such as prostate cancer. Some interventions were targeted at men, but these were not necessarily designed specifically for men, some were workplace interventions. In other words, they targeted settings or localities where men came together, such as male dominated workplaces or sports clubs. Finally, we identified only three interventions which were specifically designed with men in mind, and that can probably be called 'gender' sensitive. We would like to argue that no single one of these three approaches is necessarily better than the others, as Galdas et al. [50] reminded us "not all men are the same, nor does it make sense to assume that individual men behave similarly in all help-seeking contexts."

### Limitations of Study

The majority of studies in our review reported effective interventions. However, publication bias is a possibility, as studies with significantly positive results are more likely to be published than those with non-significant or negative results [51].

Only RCTs and controlled before and after studies were included in this review. However, we are aware that a number of process evaluations have been carried out in the UK [52-54]. We are also aware that there are published evaluations of generic interventions providing separate results for men and women, which have not been assessed in this review as they did not involve targeting men.

## Conclusion

Most of the existing evidence relates to male sex-specific services eg prostate cancer, as opposed to general services directed at problems experienced by both men and women. Of the non sex-specific interventions, only four were for men only in either content or setting. Therefore, we cannot conclude from this review that targeting men works better than delivering a general service to all people. There is little published evidence on how to improve men's uptake of services and it remains unclear whether it is more effective to provide different services or the same services in a different way.

In order to make recommendations on future gender specific services, it is important for policy-makers to appraise the effectiveness of targeting health promotion interventions at men compared with the same interventions aimed at men and women in general or at various sub-groups of the population with particular needs not necessarily related to sex or gender (deprived communities, ethnic background, age etc).

The area of men's health is still a young area of interest and good evaluations are needed to generate evidence (either way). Large-scale studies should be funded to help produce evidence that is sufficiently robust to add to the small evidence base that currently exists in this field.

## Competing interests

The authors declare that they have no competing interests.

## Authors' contributions

LMR developed and performed searches, screened search results, organised retrieval of papers, abstracted data from papers, performed data synthesis and wrote the manuscript. FD, AL and EvT were involved in drafting and revising the manuscript. AL participated in the data analysis. GR assisted in screening search results. All authors contributed to the design of the review, screened selected papers against inclusion criteria, participated in the interpretation of the data and gave final approval of the version to be published.

## Pre-publication history

The pre-publication history for this paper can be accessed here:



## Supplementary Material

Additional file 1 Search sources and strategies. Click here for file
