# Supplementary material for: What works with men? A systematic review of health promoting interventions targeting men
Source: BMC Health Serv Res. 2008 Jul 3;8:141. doi: 10.1186/1472-6963-8-141 (PMC2483970; doi:10.1186/1472-6963-8-141)
Supplement: Additional file 1 — Search sources and strategies. [file 1472-6963-8-141-S1.doc]

Search Sources and Strategies

# Sources

BNI (British Nursing Index)

Campbell Collaboration Library Catalogue

CINAHL (Cumulative Index to Nursing & Allied Health Literature)

Cochrane Controlled Trials Register

Embase

ERIC (Educational Resources Information Centre)

Health Development Agency Publications database (www.had-online.org.uk)

HEBS Library Catalogue

HMIC (Health Management Information)

MEDLINE

PsycINFO

ISI Science & Technology -- Proceedings

SCI-EXPANDED (Science Citation Index Expanded)

SSCI (Social Sciences Citation Index)

www.menshealthnetwork.org

www.emhf.org

[www.menshealthforum.org.uk](http://www.menshealthforum.org.uk/)

[www.malehealth.co.uk](http://www.malehealth.co.uk/)

[www.workingwithmen.org](http://www.workingwithmen.org/)

[www.healthofmen.com](http://www.healthofmen.com/)

# Strategies

The strategy used in the MEDLINE search is given below. This was adapted for use in the other databases.

1. (men or men's).tw.
   2. (pub or pubs).tw.
   3. (public house or public houses).tw.
   4. befriend$3.tw.
   5. buddy.tw.
   6. (call centre$1 or call center$1).tw.
   7. chat room.tw.
   8. (nightclub or nightclubs).tw.
   9. (night club or night clubs).tw.
   10. (coffee bar or coffee bars).tw.
   11. (coffee house or coffee houses).tw.
   12. (community setting$1 or community development$1 or community nurse$1 or community project$1 or community venue$1 or community worker$1).tw.
   13. exp community health nursing/ or exp community mental health services/ or exp community networks/ or exp community pharmacy services/ or exp preventive health services/
   14. (day centre$1 or day center$1).tw.
   15. detached work$3.tw.
   16. drop in clinic$1.tw.
   17. drop in service$1.tw.
   18. drop in centre$1.tw.
   19. drop in center$1.tw.
   20. (flexible opening or flexible time$1).tw.
   21. supporters club$1.tw.
   22. exp FOOTBALL/
   23. football.tw.
   24. foyer.tw.
   25. group work.tw.
   26. (health campaign or health campaigns).tw.
   27. (health day$1 or health event$1 or health stall$1 or health club$1).tw.
   28. exp Health Fairs/
   29. health awareness.tw.
   30. health check.tw.
   31. helpline$1.tw.
   32. hostel$1.tw.
   33. exp Hotlines/
   34. (information point$1 or information display$1).tw.
   35. information line$1.tw.
   36. internet.tw.
   37. website$1.tw.
   38. job club$1.tw.
   39. (leisure centre$1 or leisure center$1).tw.
   40. (male adj clinic$1).tw.
   41. exp MANUALS/
   42. male friendly.tw.
   43. (media material$1 or media campaign$1).tw.
   44. men only clinic$1.tw.
   45. (mens health clinic$1 or mens health day$1 or mens health group$1 or mens health night$1 or mens health manual$1 or mens health fair$1).tw.
   46. (mobile unit or mobile units).tw.
   47. (caravan or caravans).tw.
   48. mobile clinic.tw.
   49. mobile health units/
   50. MOT.tw.
   51. net surfing.tw.
   52. nurse led.tw.
   53. one-to-one counselling.tw.
   54. one-to-one counseling.tw.
   55. one-stop shop$1.tw.
   56. online service$1.tw.
   57. outdoor pursuit$1.tw.
   58. outdoor activit$3.tw.
   59. out of hours service$1.tw.
   60. out of hours clinic$1.tw.
   61. outreach.tw.
   62. (peer led or peer education or peer support or peer mentor$3 or peer support group$1).tw.
   63. exp peer group/
   64. probation.tw.
   65. (recreation$2 centre$1 or recreation$2 center$1).tw.
   66. (resource centre$1 or resource center$1).tw.
   67. road show$1.tw.
   68. rugby.tw.
   69. (shopping centre$1 or shopping center$1).tw.
   70. exp Soccer/
   71. social marketing.tw.
   72. supermarket$1.tw.
   73. (telephone line$1 or phone line$1 or advice line$1).tw.
   74. exp Telephone/
   75. toolkit$1.tw.
   76. (unemployment centre$1 or unemployment center$1 or job center$1 or job centre$1).tw.
   77. village hall$1.tw.
   78. walk in clinic$1.tw.
   79. (walk in centre$1 or walk in center$1).tw.
   80. web-based.tw.
   81. well men check$1.tw.
   82. well man check$1.tw.
   83. (well man$1 clinic$1 or well man$1 health clinic$1).tw.
   84. (well men$1 clinic$1 or well men$1 health clinic$1).tw.
   85. (well man$1 service$1 or well men$1 service$1).tw.
   86. well person clinic$1.tw.
   87. working mens club$1.tw.
   88. workplace$1.tw.
   89. workshop$1.tw.
   90. young offenders institution$1.tw.
   91. youth club$1.tw.
   92. mens health.tw.
   93. mens health service$1.tw.
   94. exp Health Promotion/
   95. exp Health Education/
   96. or/2-95
   97. 1 and 96
   98. limit 97 to (english language and yr=2005-2006)
   99. limit 98 to "all adult (19 plus years)"
   100. animal/
   101. human/
   102. 100 not 101
   103. 99 not 102
